# Rectal hodgkin lymphoma in a patient with ulcerative colitis: a case study

**DOI:** 10.1186/s13000-015-0271-7

**Published:** 2015-04-16

**Authors:** Simon Ladefoged Rasmussen, Christian Thomsen

**Affiliations:** Department of Gastrointestinal Surgery, Clinical Cancer Research Center, Aalborg University Hospital, Hobrovej 18-22, 9000 Aalborg, Denmark; Institute of Pathology, Clinical Cancer Research Center, Aalborg University Hospital, Aalborg, Denmark

**Keywords:** Hodgkin lymphoma, Ulcerative colitis, Immunodeficiency, Rectal tumor

## Abstract

A case of Hodgkin lymphoma located in the rectum of a patient with ulcerative colitis is described. The patient was a 44 year old male treated with thiopurines for ulcerative colitis for ten years. He was admitted with malaise, weight loss and abdominal pain. Endoscopy revealed a large ulcerative lesion involving the rectum and distal part of the sigmoid colon. Although it macroscopically resembled a rectal cancer, repeated biopsies did not reveal any malignancy. In order to resolve the symptoms of stenosis and to get the final diagnosis a recto-sigmoid resection was performed. Pathologic examination revealed nodular sclerosis classical Hodgkin lymphoma, positive for Epstein Barr Virus. Subsequent examination revealed disseminated disease involving the pelvic wall, liver, and bone marrow. The patient is currently receiving chemotherapeutic treatment, and follow-up shows disease remission.

Hodgkin lymphoma associated with immunosuppressive therapy is rare. However, patients with ulcerative colitis receiving such treatment are at increased risk of lymphoproliferative disordes, potentially due to loss of immunosurveillance and presence of oncogenic viruses (i.e. Epstein-Barr virus).

## Background

Hodgkin lymphoma (HL) is a relatively rare disease with an estimated 9.190 new cases in the United States in 2014 [1].Primary extranodal lymphomas of the gastrointestinal tract are rare. Lymphomas only account for 0.2-0.6% of large bowel malignancies [2] and primary HL involving the gastrointestinal tract is only reported in a limited number of case reports [3-5].

Most patients with HL do not have a history of immunodeficiency, but a small subset of cases arise in patients with acquired immunodeficiency (e.g. patients suffering from human immunodeficiency virus) and patients receiving immunomodulatory therapeutic agents (e.g. patients who have received organ transplants) [6,7].

In this report, a rare case of an advanced stage HL diagnosed in the rectum and sigmoid colon of patient with ulcerative colitis is presented.

## Case presentation

A 44 year old male presented in 2004 with a history of abdominal pain, intermittent diarrhoea, and rectal bleeding. A colonoscopy was performed, and severe inflammation was found in the recto-sigmoid mucosa. The clinical, endoscopic, and pathologic findings were consistent with ulcerative colitis. Initial medical treatment was Azathioprine (50 mg, three times a day), Prednisolone (50 mg, one time a day), and 5-aminosalicylic acid (800 mg, three times a day). The patient was continually treated with Azathioprine through ten years. On multiple occasions, he was admitted with exacerbations, and treated with Prednisolone and 5-aminosalicylic acid.

**Figure 1:**
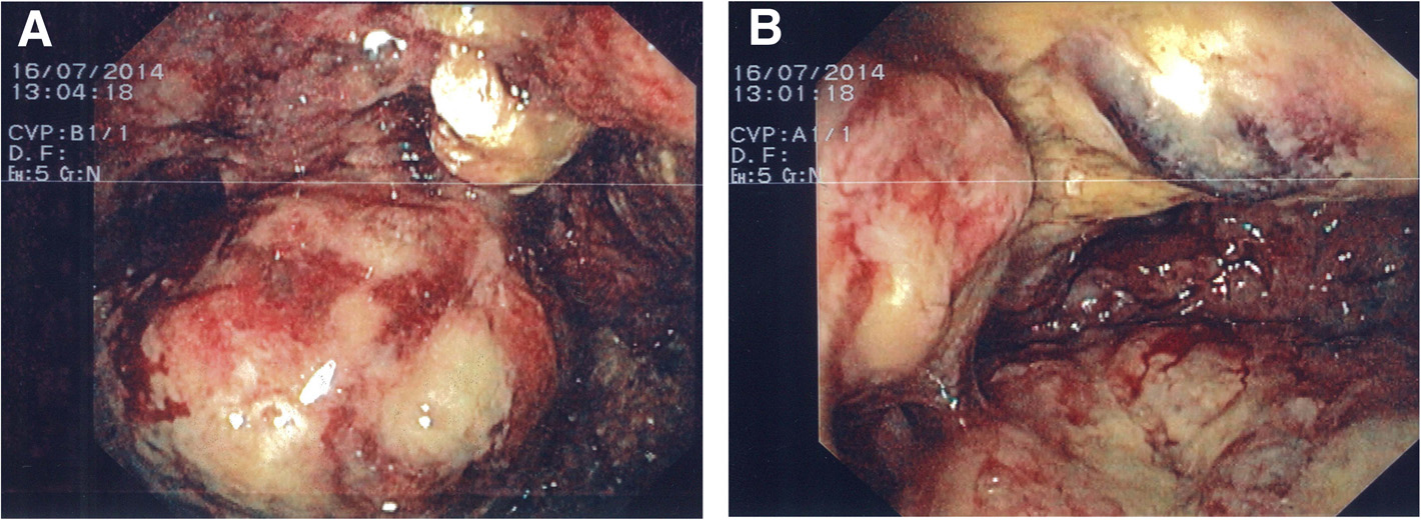
Endoscopic images of the rectum. **A)** Large ulcerative lesion located in the lower center of the image. **B)** Large ulcerative lesion located in the left side of the image.

In 2014, the patient experienced malaise, weight loss, night sweat, and intermittent fever for three to four months before admission. Sigmoideoscopy showed a large ulcerative lesion obstructing the recto-sigmoid colon (Figure 1). Multiple biopsies did not reveal malignancy but macroscopically it was believed to be a colorectal cancer. In order to resolve the symptoms of stenosis and to reveal the final diagnosis, a partial recto-sigmoid resection was performed. The workup of the patient was initially delayed by a simultaneous bilateral pulmonary embolism, for which the patient needed treatment with low molecular weight heparin.

### Pathology

**Figure 2:**
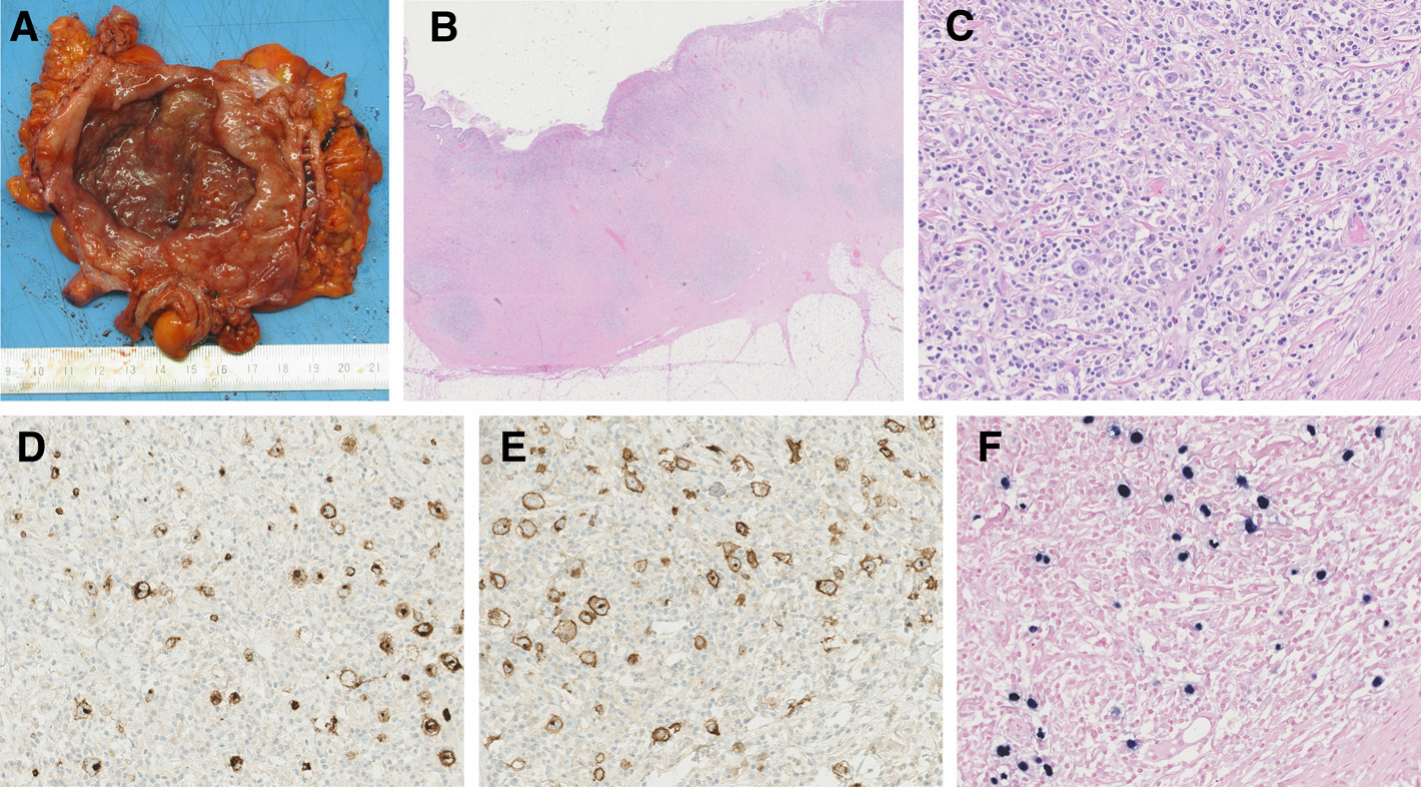
Resection specimens. **A)** Gross section image showing the opened sigmoid colon with a deep ulcer. **B)** Low power image of the gut wall showing the transition from unaffected mucosa to ulcer (notice the nodules and the marked fibrosis in the deep parts of the gut wall). **C)** High power image of a nodule showing Hodgkin and Reed-Sternberg cells in an inflammatory environment. **D)** High power image showing Hodgkin and Reed-Sternberg cells positive for CD15. **E)** High power image showing Hodgkin and Reed-Sternberg cells positive for CD30. **F)** High power image demonstrating Epstein-Barr virus by positive fluorescence in situ hybridization reaction for Epstein-Barr virus encoded small RNA’s (EBER).

The recto-sigmoid resection specimen showed a deep ulcer measuring 4 × 5 cm with relatively sharp edges (Figure 2A). Corresponding to the ulcer there was a tumorous swelling of the serosa, which was dark and granulated.

Histological evaluation of the ulcer revealed nodular sclerosis classical HL, with Hodgkin and Reed-Sternberg cells surrounded by nodular infiltrates of lymphocytes, plasma cells and eosinofilic granulocytes, in the muscularis propria and subserosa (Figure 2C). Immunohistochemically, the Hodgkin and Reed-Sternberg cells stained positive for CD15, CD30, PAX5 (weak), and fluorescence in situ hybridization for EBER (Epstein–Barr virus-encoded small RNAs) was positive (Figure 2D-F).

At the same time focal extranodal, but no nodal, involvement of the sigmoid and rectal serosa, the sigmoid mesocolon and the peritoneum of the urinary bladder was demonstrated histologically.

### Postoperative course and treatment

**Figure 3:**
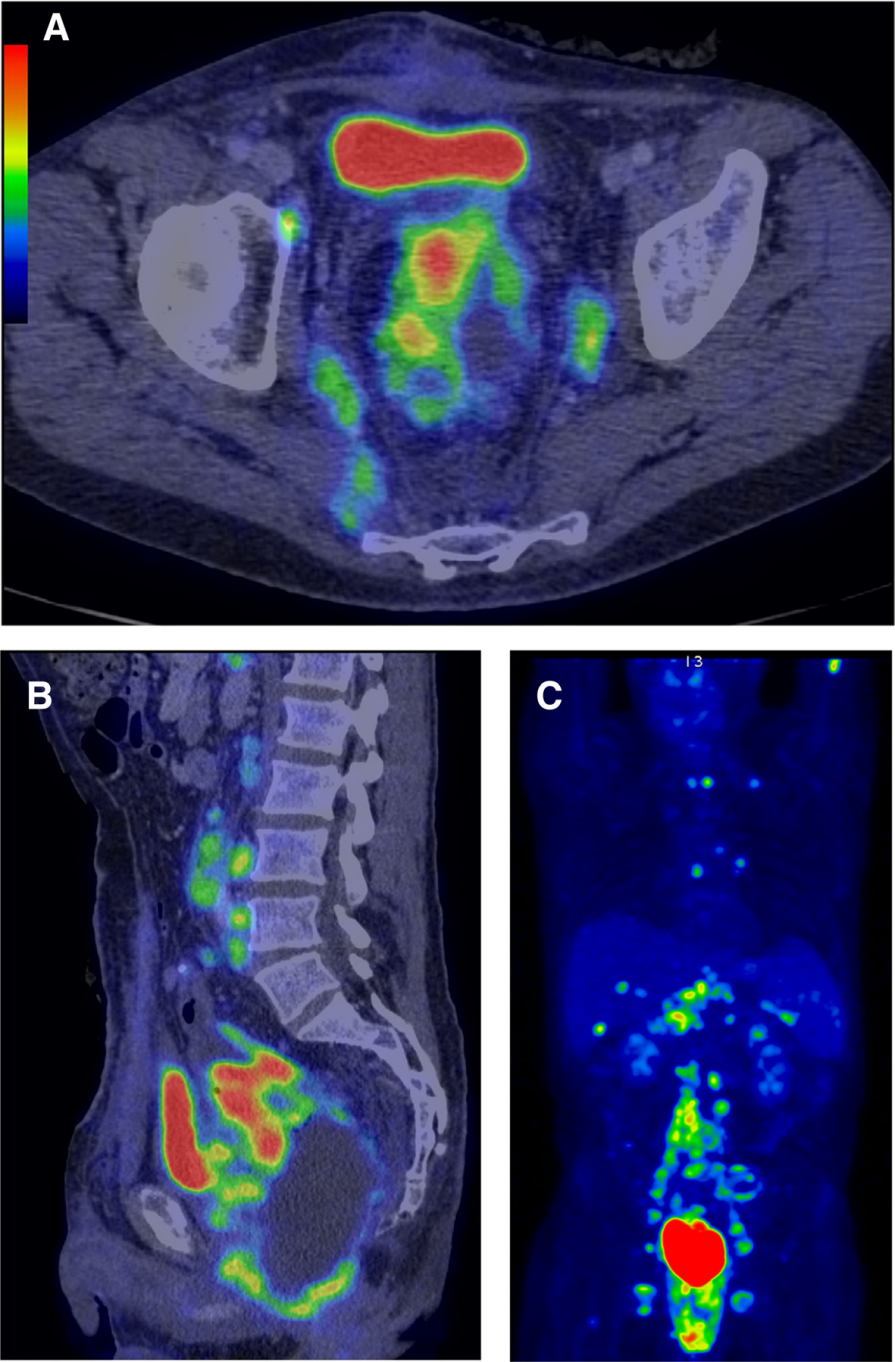
Positron emission tomography-computed tomography images. **A)** Transverse image showing increased metabolic activity in the sigmoid colon and pelvic wall. **B)** Saggittal image showing the extent of the tumor proximally in the sigmoid colon including increased metabolic activity in the pelvic wall and para-aortic lymph nodes. **C)** 3D image showing the extent of the disease, with increased metabolic activity in para-aortic lymph nodes, left axillary lymph nodes, bone marrow (T1 and T6), liver, spleen, and pelvic wall. Iliac lymph nodes are regarded as reactive.

Postoperative positron emission tomography-computed tomography (PET-CT), revealed advanced stage disease, with increased metabolic activity in the liver, pelvic muscles, and bone marrow in the thoracic region (Figure 3).

Four months after the surgery, the patient had received six of twelve rounds of chemotherapeutic treatment, with Adriamycin, Bleomycin, Vinblastine, and Dacarbazine. Follow-up PET-CT has shown near complete metabolic remission.

### Discussion

Patients with ulcerative colitis are at increased cancer risk, especially with regard to colorectal adenocarcinoma, with annual incidence rates being elevated by close to 30%, compared to patients without inflammatory bowel disease (IBD) [8]. However, a recent study was not able to reproduce this increased risk when stratifying for treatment with immunomodulating agents (including thiopurines) [9].Whether the risk of malignant lymphoma in patients with IBD is increased is controversial. Recent studies have found no, or only a marginally elevated risk of lymphoproliferative disorders [9-11]. However, it has been shown that the risk of developing lymphoproliferative disorders is increased in IBD as well as other patients receiving thiopurines as treatment (HR, 5.28; 95% CI, 2.01-13.9) [12]. An increased incidence of lymphomas in IBD patients could, therefore, be attributed to treatment with immunosuppressive drugs. A study on patients with rheumatoid arthritis showed that high inflammatory activity was a risk factor for lymphoma development (OR, 5.8; 95% CI, 3.1-213) [13], and it has been speculated that the same could be the case in patients with ulcerative colitis [14].

HL attributed to treatment with immunosuppressive drugs is very rare and only described in case studies. These cases are primarily described in patients with rheumatoid arthritis and Crohn’s disease, promoting IBD patients treated with immunosuppressants as a group a higher risk [15]. The majority of cases described were also positive for Epstein-Barr virus promoting oncogenic viral infection as an additional risk factor [15].

The diagnosis of extranodal HL can be a challenge. Classical HL is based on the identification of mononucleated Hodgkin cells and multinucleated giant cells termed Reed-Sternberg cells, within an inflammatory environment [16]. The Hodgkin and Reed-Sternberg cells are however, vastly outnumbered by reactive inflammatory cells, which constitute from 90–99.9% of the tumour mass [16].

In the present case, the initial biopsies only showed chronic ulcer, while surgical resection provided material from the deeper parts of the gut wall, which revealed the final diagnosis. The preoperative biopsies were reviewed with the disease in mind, but no Reed-Sternberg cells could be distinguished.

## Conclusions

The patient had received treatment with both thiopurines (continually) and 5-aminosalicylic acid (intermittently) through ten years. Special attention should be given to such patients, when they present symptoms that are not characteristic of their known inflammatory disease, as their risk of lymphoproliferative disorders is increased. Even though extranodal presentation of HL is a rare entity, the possibility should be kept in mind, as survival relies on early and efficient treatment.

## Consent

Written informed consent was obtained from the patient for publication of this Case Report and any accompanying images. A copy of the written consent is available for review by the Editor-in-Chief of this journal.

## Abbreviations

HL: Hodgkin lymphoma
PET-CT: Postoperative positron emission tomography-computed tomography
IBD: Inflammatory bowel disease
